# Opportunities to reduce the environmental impact of endoscopy: a detailed material flow analysis of diagnostic colonoscopy

**DOI:** 10.1055/a-2773-5144

**Published:** 2026-02-17

**Authors:** Ceyda B. Izci, Maria Sofia Clercx Lao, Britta Vegting, Peter D. Siersema, Maike V. Tietschert, Nicole G.M. Hunfield, Jan-Carel Diehl, Pieter Jan F. de Jonge

**Affiliations:** 1 Gastroenterology and Hepatology 6993 Erasmus MC University Medical Center RotterdamRotterdamNetherlands; 2 Design for Sustainability 2860 Delft University of TechnologyDelftNetherlands; 3 Socio-Medical Sciences 113896 Erasmus Universiteit Rotterdam Erasmus School of Health Policy and ManagementRotterdamNetherlands; 4 Adult Intensive Care 6993 Erasmus MC University Medical Center RotterdamRotterdamNetherlands; 5 Hospital Pharmacy 6993 Erasmus MC University Medical Center RotterdamRotterdamNetherlands

## Abstract

**Background:**

Gastrointestinal endoscopy is the third largest contributor to hospitals’ environmental footprints. Colonoscopies, among the most frequently performed endoscopic procedures, are considered to have a significant environmental footprint, yet their exact impact is unknown. We analyzed the material flow of diagnostic colonoscopy to identify opportunities for reducing its environmental impact.

**Methods:**

A single-center material flow analysis (MFA) was conducted during a week with diagnostic colonoscopy procedures. Waste was collected, weighed, and categorized by product type. The material composition was identified. Using visual mapping, we identified which products were used and discarded at each stage of the procedure.

**Results:**

15 diagnostic colonoscopy procedures were audited. Mean (SD) waste mass per procedure was 0.58 (0.01) kg, including an average of 46 products. Extrapolated to the hospital’s annual diagnostic colonoscopies, this equals approximately 1169 kg of waste. Plastic-based products constituted the largest product category (22.7%). Nitrile made up the largest material category (14.1% of the total weight of materials). The estimated carbon footprint for the analyzed waste was 1.77 kg CO _2_ e, with nitrile and polypropylene being the largest contributors. Most products were used and discarded in the preparation stage of the colonoscopy procedure.

**Conclusions:**

Plastic-based materials were the largest contributor to the total used product weight. These findings inform decision-makers in designing targeted interventions to reduce the environmental impact of diagnostic colonoscopy, for example by considering more sustainable materials such as fiber-based materials. The prepared visual map makes waste generation discussable among healthcare professionals, thereby supporting a collaborative approach to identify opportunities to improve sustainability.

## Abstract (de)

**Graphical Abstract:**

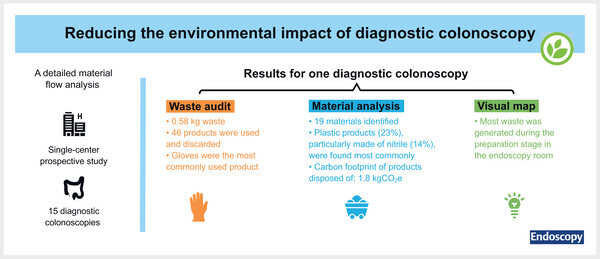

## Introduction

The Dutch healthcare sector contributes approximately 7% to the total national CO _2_ emissions, produces 4% of total waste, and extracts 13% of raw materials 1 . Within hospitals, endoscopy departments have been identified as the third largest contributor to the environmental impact of hospitals 2 . The primary drivers are a high volume of endoscopy procedures, the intensive cleaning process to enable the reuse of endoscopes, extensive use of single-use medical equipment and consumables, and patient travel 1 .

In 2024, approximately 159000 colonoscopies were performed in the Netherlands 3 . Because of the high frequency of this procedure, colonoscopies are likely to represent a major source of an endoscopy department's waste. Previous studies have taken multiple methodological approaches to estimate the waste generated by colonoscopy procedures, and have produced varying results. Across these studies, however, esophagogastroduodenoscopy (EGD) and colonoscopy procedures have been identified as contributing significantly to waste. For example, studies from France and the USA combined data from EGD and colonoscopy procedures, reporting average waste generation of 1.5 kg per procedure in France and 3.1 kg in the USA 4 5 .

Current studies do not however provide a detailed breakdown of the types of waste associated with colonoscopy or their material composition. Moreover, none of these studies have examined the specific stages of the procedure at which waste is generated. Understanding how much waste is generated during which part of the procedure and the material composition of this waste is crucial for developing interventions and innovative solutions that can reduce the environmental impact of endoscopy departments.

For a comprehensive analysis of these three parameters, we conducted a detailed material flow analysis (MFA). In contrast to other environmental assessment methods, such as life cycle assessment (LCA), which take a wider approach, MFA provides the granularity needed to inform targeted waste-reduction strategies.

Accordingly, we systematically quantified the amount of waste generated by diagnostic colonoscopy procedures at a university hospital. The waste was categorized into different product categories, and the amount of each product type was quantified; these data were then used to calculate the carbon footprint of the material composition. Finally, we created a visual map to identify when waste was disposed of during the specific stages of the procedure. Based on the results, we provide suggestions for reducing the environmental impact of colonoscopy procedures.

## Methods

### Study design and scope

This prospective study was conducted in February 2024 at the endoscopy department of the Erasmus MC University Medical Center (MC) Rotterdam, The Netherlands. Written informed consent was not required because this study did not involve the collection or use of any personal medical data. Waste generation was not linked to any patient information, and the endoscopy procedure remained undisturbed.

An MFA, a systematic assessment method to quantify the flows of materials within a system 6 , was used to identify the materials used and disposed of during diagnostic colonoscopy. Unlike an LCA, which evaluates environmental impacts across the entire life cycle of a product or a process, MFA focuses on the detailed quantification and mapping of material use and waste generation within a specific process. This granularity makes MFA particularly suitable for identifying concrete waste-reduction opportunities in the clinical workflow. In our study, MFA was applied with waste mass in kg as the primary metric. Our method aligns with the framework of the Endoscopic Sustainability Primary Reporting Essentials (E-SPARE) position statement 7 by systematically categorizing material flows.

The MFA was conducted in three stages: (1) a waste audit to systematically collect and estimate the solid general waste generated from diagnostic colonoscopy (this included measuring the mass of the waste, counting the number of products used, and categorizing them by product type; (2) analysis of each product to determine its material composition; (3) observations of diagnostic colonoscopy procedures to identify which products were discarded at which stage of the procedure.

The MFA was conducted for diagnostic colonoscopy procedures, as these represent the majority of colonoscopies performed in the endoscopy department that we studied. We included patients referred for diagnostic indications, such as rectal bleeding, polyp surveillance, or diarrhea, and procedural interventions, including taking biopsies and/or performing endoscopic mucosal resection (EMR) for polyps <20 mm. Therapeutic colonoscopies, such as those involving endoscopic submucosal dissection (ESD), stent deployment, or endoscopic full-thickness resection (EFTR), were excluded from this study as they represent only a small proportion (<5%–10%) of all colonoscopy procedures in most endoscopy units.

Procedures were performed either without sedation or with mild sedation using midazolam and/or fentanyl. The waste stemming from the medication that was administered in the hospital as part of the procedure was included in the analysis. Laxatives used at home during the preparation phase of the colonoscopy were excluded. All products used and disposed of during diagnostic colonoscopy procedures were documented and included in the MFA. These items comprised both universally required products in the endoscopy room, such as gloves and aprons, and procedure-specific products, such as biopsy forceps and polyp traps ( Table 1 ).

**Table:** **Table 1** Products included in the material flow analysis.

Endoscopy room products	Colonoscopy-specific products
Gloves	Disposable dignity shorts
Compresses	Sterile lubricant
Syringes	Formalin pots
Paper and sterile tissues	Forceps and snares
Needles	Polyp traps
Aprons	
Kidney dishes	
Ampoules	
Waste bags	
Waterjet connectors	
Absorbent fiber mats	
Intravenous butterfly catheters	
Disposable oxygen tubes	
Disposable wash cloths	
Tubing	
Sterile water bottles	

### Waste audit

A waste audit was conducted over a 1-week period, which consisted of 5 working days. In this study, hazardous chemicals were defined as bodily fluids and residual medicines; handling and quantifying these outside the established disposal procedure would have posed contamination risks, and therefore these were excluded from the waste audit. Waste bags containing solid waste from colonoscopy procedures in the endoscopy room were collected, and the number of colonoscopy procedures performed each day was documented. The total weight of the waste bags was measured using a hanging scale (Kern CH15K20, Germany) with a precision of 0.01 g. These measurements were recorded to allow calculation of the mean weight of waste generated by a single colonoscopy procedure.

The solid waste was manually sorted by two researchers wearing standard personal protective equipment (PPE). Waste was categorized into six product categories: paper-based products, absorbent products, plastic-based products, metal composite products, sterile packaging, and PPE. Products were placed by category into separate crates and weighed using a precision scale (Kitchenwell KN353, The Netherlands) with an accuracy of 1 g. After these measurements had been recorded, the contribution of each product category to total waste was calculated as a percentage. Additionally, the number of individual products retrieved from the waste bags was counted and documented to calculate the average amount of products used per procedure.

### Material analysis

Analysis of the material composition of each discarded product was conducted, followed by measuring the weight of each identified material type. The data collection process for the material analysis consisted of five steps: (1) each product was separated from its packaging; (2) the product was weighed; (3) the different material types within each product were identified and separated (for example, in the case of a needle, the metal component was manually removed by cutting it from the plastic handle); (4) each material component was individually weighed; (5) desk research was conducted to determine the material type of each component, for example polyvinylchloride (PVC) or high-density polyethylene (HDPE). When weighing the products and components (steps 2 and 5), one of two balances were used: for >10 g, a digital scale (Revall Digital, China) with a precision of 0.1 g, and for <10 g, a kitchen scale (Kitchenwell KN350) with a precision of 0.01 g.

To estimate the carbon footprint equivalent of the materials, emission factors (kg CO _2_ e per kg of material) were obtained from the Ecoinvent v3.10 database 8 , using the IPCC 2021 GWP100 method for global warming potential characterization. The assessment followed a cradle-to-grave approach, accounting for the impact from raw material extraction to disposal. For each identified material type, the corresponding emission factor was multiplied by its measured weight in kg to calculate the estimated carbon footprint equivalent. This allowed the material composition data to be translated into a carbon footprint equivalent (in kg CO _2_ e) for each material type.

### Visual mapping of diagnostic colonoscopy procedures

The visual mapping method offers opportunities to collaboratively explore interventions with nurses, aiming to reduce environmental impact across the different stages of the colonoscopy procedure. This method was chosen because visual representations can translate complex systems into concrete forms, making it easier to envision future solutions 9 10 11 . Additionally, the visual map contributes to a shared understanding of the system among endoscopy staff and other stakeholders.

With input from clinicians, the researcher developed a visual map illustrating the colonoscopy process – from the preparation of the endoscopy room to cleaning the room after the procedure. Based on observations made during the 1-week period, the researcher identified and placed all relevant products on the map to show how and in which stage each was used. To validate the accuracy of the map, it was subsequently reviewed by two nurses. This resulted in the development of its final version.

## Results

### Waste audit

In 2024, 2016 diagnostic colonoscopies were performed at the endoscopy unit of the Erasmus MC. During the 1-week study period, 15 diagnostic colonoscopies were audited. The colonoscopy procedures generated a mean (SD) of 0.58 (0.01) kg of solid waste per procedure. When extrapolated to all diagnostic colonoscopies performed that year at the department, the estimated total solid waste was 1169 kg. All waste was collected as general waste and subsequently incinerated.

**Fig. 1:**
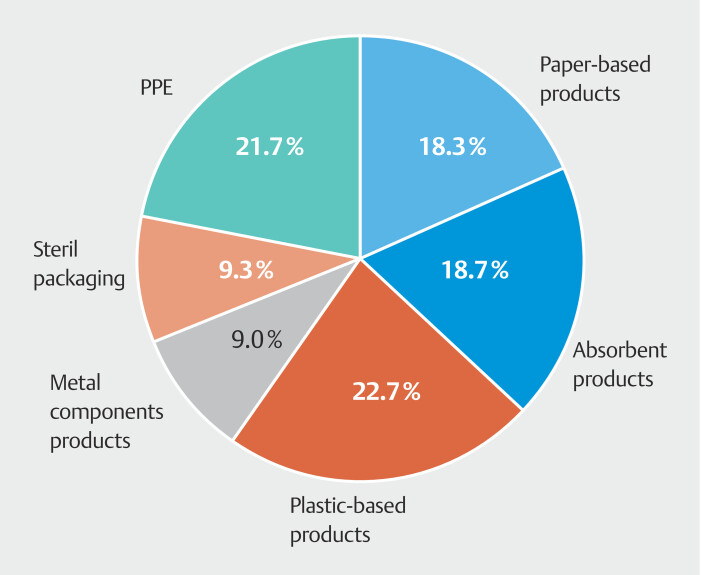
Distribution of the six product categories. PPE, personal protective equipment.

Plastic-based products and PPE were the largest product categories with 22.7% and 21.7% respectively, followed by absorbent products (18.7%) and paper-based products (18.3%) ( Fig. 1 ).

Per procedure, a mean of 46 products were used and disposed of after a single use. When extrapolated to the annual volume of diagnostic colonoscopies in the Erasmus MC, this amounts to approximately 92700 products per year.

**Fig. 2:**
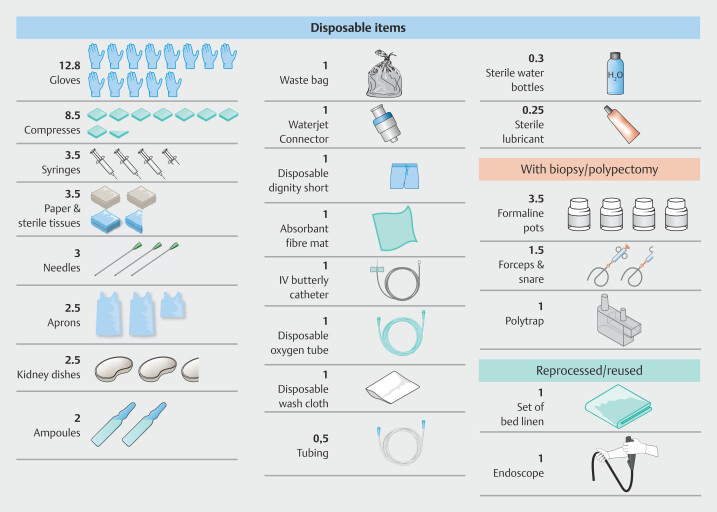
Average use of products per colonoscopy procedure.

A total of 21 different product types were identified ( Fig. 2 ). The most frequently used products were gloves (n = 12.8), compresses (n = 8.5), syringes (n = 3.5), and paper tissues (n = 3.5). This information is also provided in tabular form in **Table 1s** , see online-only Supplementary methods.

Each diagnostic colonoscopy procedure was performed by a team of three healthcare professionals (one endoscopist and two nurses). Furthermore, by dividing the total number of products used per procedure by the number of healthcare professionals involved, it was estimated that approximately four gloves were used per healthcare professional per procedure.

### Material composition

**Fig. 3:**
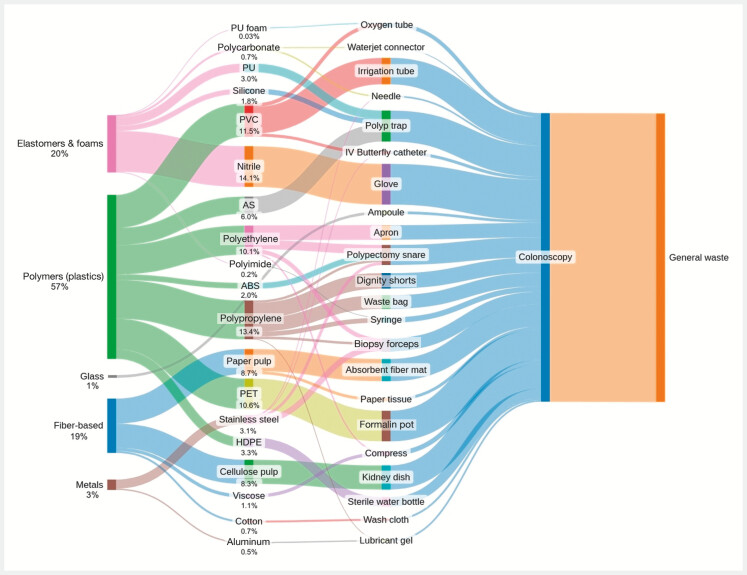
Material flow analysis of colonoscopy procedures. ABS, acrylonitrile butadiene styrene; AS, acrylonitrile styrene; HDPE, high density polyethylene; IV, intravenous; PET, polyethylene terephthalate; PU, polyurethane, PVC, polyvinylchloride.

In total, 19 different material types were detected in the waste generated by colonoscopy procedures ( Fig. 3 ). Only one product contained information about its material composition on its packaging. For the remaining products, information about their material composition was not explicitly provided. Research was conducted to retrieve the available online technical data sheets from each supplier. For certain products, suppliers were contacted to obtain the information required for the material analysis.

Fig. 3 visualizes the MFA results and details the distribution of material types across the products used and discarded for one colonoscopy procedure. The weight of the material types and the subdivisions of materials across each product are shown in **Table 2s** .

Categorizing the materials showed that 57% of the total weight of the products consisted of plastic-based materials, 20% elastomers and foams, 19% fiber-based materials, 3% metals, and 1% glass. **Table 3s** shows the weight of each material category. The most prevalent material type was nitrile, accounting for 14.1% of the total weight of all products, which was followed by polypropylene (13.4%), PVC (11.5%), polyethylene terephthalate (PET; 10.6%), polyethylene (10.1%), paper pulp (8.7%), and cellulose pulp (8.3%).

In total, the estimated carbon footprint equivalent of all analyzed waste amounted to 1.77 kg CO _2_ e ( Table 2 ). Nitrile and polypropylene were the largest contributors, each accounting for 0.28 kg CO _2_ e (15.8%), and together representing 31.6% of the total estimated carbon footprint of all materials.

**Table:** **Table 2** The carbon footprint of each material type

Material	Carbon footprint, kg CO _2_ e
Nitrile	0.28
Polypropylene	0.28
Polyvinylchloride	0.24
Polyethylene	0.20
Acrylonitrile styrene	0.16
Polyurethane	0.12
Stainless steel	0.10
PET	0.06
HDPE	0.06
ABS	0.06
Silicone	0.04
Paper pulp	0.03
Polycarbonate	0.03
Cotton	0.03
Cellulose pulp	0.02
Viscose	0.02
Aluminum	0.02
Glass	0.01
Polyisoprene	0.005
Polyurethane foam	0.001
**Total**	**1.77**
ABS, acrylonitrile butadiene styrene; HDPE, high density polyethylene; PET, polyethylene terephthalate.	

### Visual map of the colonoscopy procedure

**Fig. 4:**
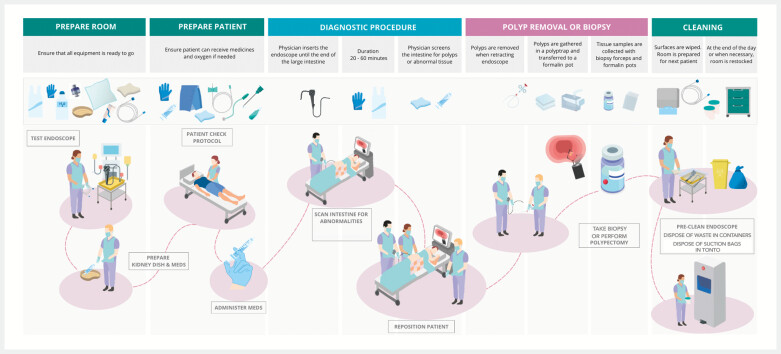
Visual map of the products used and disposed of at each stage of the colonoscopy procedure.

A visual map was developed to provide a clear overview of the products used and disposed of throughout the different stages of the colonoscopy procedure ( Fig. 4 ). Icons were used within the visual map to indicate which products were used at each stage. Based on our observations, the colonoscopy procedure was divided into five stages: preparation of room, patient preparation, diagnostic procedure, polyp removal or biopsy, and cleaning.

Most of the waste was generated during the preparation stage of the endoscopy room. The products contributing most to the total weight of waste were nitrile gloves, which healthcare professionals start wearing during the preparation of the room stage. The second largest contributor to the overall weight of waste was dignity shorts made of polypropylene, which patients wear during the preparation stage.

## Discussion

By conducting an in-depth analysis of associated product types and amounts, material composition, and the stage of the procedure in which each product was disposed, the waste audit concluded that each diagnostic colonoscopy generated 0.58 kg of waste, totaling 1169 kg annually at the Erasmus MC. The high frequency of procedures results in a considerable weight of overall waste, so highlighting the importance of identifying opportunities for waste reduction.

On average, 46 products were used during each procedure, with plastic-based materials accounting for 57% of the total product weight and with nitrile being the most frequently used material (14.1%). Additionally, the visual map ( Fig. 4 ) illustrates which products were being disposed of at each stage of the procedure, revealing that most of the waste is generated during the stage of room preparation.

Several studies have quantified the waste generated by endoscopic procedures, each using different methodologies, which limit comparability across studies and settings. For example, studies that measured the total weight of waste for an endoscopic procedure reported higher numbers, such as 3.1 kg and 1.5 kg per procedure 4 5 . Another study reported a carbon footprint of 6.71 kg CO₂ per colonoscopy procedure, but the lack of corresponding waste weight data complicates comparing these 12 . A study that defined what was included in its waste measurement reported 0.9 kg of waste per colonoscopy, including 0.4 kg of landfill waste 13 . This figure is comparable to our findings with an average of 0.58 kg of waste per procedure. However, designing targeted strategies for reducing the environmental impact of colonoscopy procedures requires an understanding of the whole system, instead of a single number. Therefore, our MFA takes a detailed approach by analyzing the types of products used, their material composition, and the specific stages at which they were discarded.

Our MFA demonstrated that on average 19 different material types were used for all products. This large variety complicates the recycling process, as different material types require separate handling and processing methods. Plastic-based materials were the largest category, making up 57% of the total weight of the products. The commonest material was nitrile, compromising 14.1% of the total weight of all products, which was followed by polypropylene (13.4%), PVC (11.5%), PET (10.6%), polyethylene (10.1%), paper pulp (8.7%), and cellulose pulp (8.3%).

The main contributors to the total carbon footprint of the material composition, amounting to 1.77 kg CO₂e, were nitrile and polypropylene, with each accounting for 0.28 kg CO _2_ e and together representing 31.6% of the total. Their high contribution results from their extensive use and moderate emission factors ( **Table 4s** ). Reducing their quantities or replacing nitrile and polypropylene with bio-based or recycled polymers could lower the overall carbon footprint. For example, nitrile is derived from fossil fuels, requires significant energy during its production, and is nonbiodegradable 14 . Studies into bio-based or biodegradable polymers, including natural rubber latex from renewable sources, as well as novel compostable polymers such as polyhydroxyalkanoates (PHAs), have shown potential as more sustainable alternatives 15 . These options are however not widely available currently, and most are yet to be certified for clinical use. Cellulose and paper pulp, identified in the material composition analysis, are fiber-based renewable materials, so offer a more sustainable alternative. Increasing the use of these alternatives could further enhance sustainability efforts and reduce plastic waste.

Another remarkable finding of our study was that only one product contained a label with a detailed material composition. Healthcare professionals therefore have limited information on the material composition of the products that they use. Instead, extensive desk research was required to find technical data sheets for the remaining products and, in some cases, suppliers needed to be contacted to inquire about information on their material composition. More transparency from suppliers regarding the materials composition of their products is essential to improve waste management in the endoscopy room. Clearly labelling the material on the product or packaging may enhance recycling as it makes it easier to sort and process waste appropriately.

Certain products and packaging should be considered for redesign to enhance sustainability. Our MFA revealed several insights into the current design of products and packaging. For example, incorporating reusable components, such as handles on biopsy forceps or polyp snares, could significantly reduce the waste generated by these products.

In some cases, packaging accounted for a large portion of the product's total weight, as observed with waterjet connectors. This raises questions as to whether packaging could be optimized by bundling products together, thereby reducing overall packaging waste. Additionally, many packaging designs use a combination of plastic and paper, which complicates recycling owing to the need for material separation. By minimizing excess packaging and using one material for packaging, manufacturers can support sustainable waste management practices. Lastly, product information provided on paper could be replaced by electronic product information to reduce waste.

The visual map that was created showed that most products are used and disposed of during the preparation stages for the colonoscopy procedure. This finding underscores the importance of focusing on waste reduction at this specific stage. Beyond serving as a documentation tool, the visual map also fostered active engagement among nurses. Its strength lies in making waste generation both tangible and discussable among healthcare professionals, thereby supporting a collaborative approach to identify sustainable opportunities.

Our study has several strengths. First, it offers an in-depth MFA of colonoscopy procedures by identifying the specific product types and amounts, their material composition, and insights into the stage of the procedure in which each product was discarded. Second, the study goes beyond quantitative analysis by incorporating qualitative research through the development of a visual map. This map was co-created and reviewed in collaboration with nurses from the green team, fostering reflection and raising awareness about waste generation throughout the procedure.

Building on these strengths, it is important to clarify how MFA compares with other environmental assessment methodologies, such as LCA, to better contextualize the scope and implications of our findings. MFA and LCA are complementary but distinct. MFA quantifies material streams within a defined system, providing a detailed breakdown of material consumption and waste by product type, and procedural stage. In contrast, LCA evaluates the environmental impacts of a product across its entire life cycle, from raw material extraction to disposal, and reports outcomes in impact metrics such as kg CO _2_ e. In our study, MFA was applied with waste mass in kg as the primary metric, enabling us to identify specific waste “hotspots” within colonoscopy procedures. While LCA offers a broader system-level perspective, MFA provides the granularity needed to inform targeted waste-reduction strategies.

Some limitations do however exist. First, this study focused only on diagnostic colonoscopy procedures, which limits the generalizability of the results to other types of endoscopy procedures. A total of 15 procedures were audited during the waste audit; however, extending data collection to additional days would be unlikely to significantly affect the results, given the minimal variation in waste weights observed across procedures (a range of only 0.01 kg). Variation is limited because diagnostic colonoscopy procedures typically involve standardized product usage. The generalizability of the waste audit outcomes to other centers or clinical contexts should be interpreted with caution, as variations in outcomes may arise from differences in staffing requirements per endoscopy procedure, local practice patterns, and hospital type (e.g. teaching vs. nonteaching institutions).

While our MFA focused on the environmental impact of waste, a complementary economic evaluation would also be valuable. Future studies should assess both the financial and environmental implications to guide effective decision-making. Furthermore, this study focused only on physical product waste. Future research could also consider other streams, such as electricity and water, to gain more perspectives on the environmental impact of colonoscopy procedures.

For the material composition analysis, we relied on technical data sheets provided by the suppliers, which we understand were based on standardized material testing methods used in product certification and regulatory compliance. While we did not conduct independent laboratory verification, we believe that the supplier data represent accurate and reliable sources as this is mandatory for the product certification.

This study focused on quantifying and characterizing the waste component of the diagnostic colonoscopy procedure. Other contributors to the overall carbon footprint, such as patient and staff transport, energy use, and equipment reprocessing, were not included within the scope of this analysis. Estimating the relative contribution of waste compared to these other factors would provide valuable context for prioritizing sustainability efforts. To this end, we have initiated work to identify emission factors and calculate the carbon footprint for the materials used in a diagnostic colonoscopy procedure. This will enable future research to assess the proportion of the carbon footprint attributable to waste in relation to other components.

In conclusion, this study highlights the importance of understanding the waste generated by medical procedures as a crucial step toward identifying opportunities for sustainability. Our findings highlight key areas for sustainable interventions:

- creation of awareness among healthcare professionals through visual mapping
- identification of material composition of the products used during diagnostic colonoscopy to explore opportunities for more sustainable material alternatives
- promotion of greater transparency from suppliers regarding the materials used in products to improve waste management practices in the endoscopy room
- redesign of products to make specific components reusable, which will reduce the amount of waste generated
- minimization of the amount of packaging waste by combining multiple products into a single package
- use of only one material for packaging to simplify recycling and contribute to more effective waste management.
- the use of electronic product information instead of paper.

## Green stamp explanation

Endoscopic procedures represent a significant environmental impact. This manuscript by identifying plastic-based products as the most relevant factor in the environmental impact of colonoscopies may enable changes in process that promote more sustainable endoscopy services. A further understanding of manufacturing and the usage of medical devices may play a role in reducing the carbon footprint of healthcare.

## Supplementary Material

Supplementary Material Zusatzmaterial
