# Supplementary material for: Opportunities to reduce the environmental impact of endoscopy: a detailed material flow analysis of diagnostic colonoscopy
Source: Endoscopy. 2026 Feb 17;58(6):618–26. doi: 10.1055/a-2773-5144 (PMC13305572; doi:10.1055/a-2773-5144)
Supplement: Supplementary file 1 — Supplementary Material [file 10-1055-a-2773-5144_27940875.pdf]

Supplementary material

Opportunities to reduce the environmental impact of endoscopy: a detailed material flow analysis of diagnostic colonoscopy

Table 1s: Weight in grams of the material types for each product

	PE	PET	HDPE	PP	PVC	PC	PI	Nitrile	PU	PU foam	ABS	AS	Silicone	Stainless steel	Paper pulp	Cellulose pulp	Cotton	Viscose	Glass	Aluminium
Needle						3								0,4						
Absorbent fiber mat															46					
Oxygen tube					7,6					0,2										
Irrigation tube					55															
Polyp trap									17,8			35,8	10,7							
IV Butterfly catheter					6,1									0,1						
Biopsy forcep	8,8			5,2										12,4						
Polypectomy snare	18,9			5							11,8			5,9						
Compress	2,9																	6,8		
Ampoule																			5,5	
Kidney dish																50				
Dignity short				31,7																
Apron	30																			
Waste bag				28,2																
Wash cloth																	4			
Paper tissue															6					
Glove								84,5												
Formaline pot		63,4																		
Sterile water bottle			19,9																	
Waterjet connector						1,3														
Lubricant gel				0,6																2,8
Syringe				9,7			1,4													
**Total weight (g)**	60,6	63,4	19,9	80,4	68,7	4,3	1,4	84,5	17,8	0,2	11,8	35,8	10,7	18,8	52	50	4	6,8	5,5	2,8

ABS, acrylonitrile butadiene styrene; AS, acrylonitrile styrene; HDPE, high density polyethylene; PC, polycarbonate; PE, polyethylene; PET, polyethylene terephthalate; PI, polyimide; PP, polypropylene; PU, polyurethane; PVC, polyvinylchloride.

Table 2s: Weight (in grams) and percentage of each material category

Material category	Weight (g)	Percentage
Polymers (plastics)	340.6	57%
Elastomers & foams	118.9	20%
Fiber-based	112.8	19%
Glass	5.5	1%
Metals	21.6	3%

Table 3s: Average use of products used per colonoscopy procedure

Products	Amount
Gloves	12.8
Compresses	8.5
Syringes	3.5
Paper & Sterile tissues	3.5
Needles	3
Aprons	2.5
Kidney dishes	2.5
Ampoules	2
Waste bag	1
Waterjet connector	1
Disposable dignity short	1
Absorbent fiber mat	1
IV butterfly catheter	1
Disposable oxygen tube	1
Tubing	0.5
Sterile water bottles	0.3
Sterile lubricant	0.25

Table 4s: The carbon footprint of each material type with weights and emission factors

Material	Weight [kg]	Emission factors [kg CO_2/kg]	Carbon footprint [kg CO_2e]
Nitrile	0.085	3.33	0.28
Polypropylene	0.080	3.52	0.28
PVC	0.069	3.45	0.24
Polyethylene	0.061	3.25	0.20
Acrylonitrile styrene	0.036	4.32	0.16
Polyurethane	0.018	6.78	0.12
Stainless steel	0.019	5.10	0.10
PET	0.063	0.95	0.06
HDPE	0.020	3.10	0.06
ABS	0.012	4.64	0.06
Silicone	0.011	3.68	0.04
Paper pulp	0.052	0.49	0.03
Polycarbonate	0.004	6.86	0.03
Cotton	0.004	8.58	0.03
Cellulose pulp	0.050	0.39	0.02
Viscose	0.007	3.33	0.02
Aluminium	0.003	7.60	0.02
Glass	0.006	2.57	0.01
Polyisoprene	0.001	3.32	0.005
PU foam	0.0002	6.94	0.001
Total			1.77

ABS, acrylonitrile butadiene styrene; HDPE, high density polyethylene; PET, polyethylene terephthalate; PVC, polyvinylchloride.
